# Pazopanib: Evidence review and clinical practice in the management of advanced renal cell carcinoma

**DOI:** 10.1186/s40360-018-0264-8

**Published:** 2018-11-26

**Authors:** María José Méndez-Vidal, Áurea Molina, Urbano Anido, Isabel Chirivella, Olatz Etxaniz, Eva Fernández-Parra, Marta Guix, Carolina Hernández, Julio Lambea, Álvaro Montesa, Álvaro Pinto, Silverio Ros, Enrique Gallardo

**Affiliations:** 1 Oncology Department, Maimonides Institute of Biomedical Research (IMIBIC), Reina Sofia Hospital, Córdoba, Spain; 2 Oncology Department, Complejo Hospitalario Universitario A Coruña, ACoruña, Spain; 3 Oncology Department, Complejo Hospitalario Universitario de Santiago, Santiago de Compostela, Spain; 4 Oncology Department, Hospital Clínico Universitario de Valencia, Valencia, Spain; 5 Oncology Department, Hospital Universitari Germans Trias i Pujol, Badalona, Spain; 6 Oncology Department, Hospital Nuestra Señora del Valme, Sevilla, Spain; 7 Oncology Department, Hospital del Mar, Barcelona, Spain; 8 Medical Oncology Department, Hospital Universitario Nuestra Señora de Candelaria, Santa Cruz de Tenerife, Spain; 9 Medical Oncology Department, Hospital Clínico Universitario Lozano Blesa, Zaragoza, Spain; 10 Medical Oncology Department, Hospital Regional de Málaga, Málaga, Spain; 11 Medical Oncology Department, Hospital la Paz, Madrid, Spain; 12 Oncology Department, Hospital Universitario Virgen de la Arrixaca, Murcia, Spain; 13 Oncology Department, Parc Taulí Hospital Universitari. Institut d’Investigació i Innovació Parc Taulí I3PT. Universitat Autònoma de Barcelona, Sabadell, Barcelona Spain

**Keywords:** Antineoplastic agents, Carcinoma, renal cell, Kidney neoplasms, Protein kinase inhibitors, Quality of life, Pazopanib

## Abstract

**Background:**

Pazopanib is indicated in the first-line treatment of metastatic renal cell cancer (mRCC). The aim of this study was to review the efficacy, safety, and pharmacokinetics of pazopanib and see how these aspects are linked to clinical practice.

**Methods:**

A non-exhaustive systematic review was conducted according to the three topics. No publication restrictions were imposed and the selected languages were Spanish and English. After that, a summary of the main results and findings of the review was presented and discussed during three meetings (one for each topic) with 13 medical oncologists that usually treat mRCC. At these meetings, a questionnaire on the first-line use of pazopanib in clinical practice was also drawn up. After the meetings, the questionnaire was completed by 60 specialist medical oncologists in renal cancer.

**Results:**

The efficacy and safety of pazopanib have been demonstrated in several clinical trials, and subsequently confirmed in studies in real-world clinical practice. In addition to its clinical benefit and good safety profile, quality of life results for pazopanib, which compare favorably to sunitinib, make it a good option in the first-line treatment of patients. Special populations have been included in studies conducted with pazopanib, and it is safe for use in elderly patients, poor functional status, kidney failure, and mild or moderate hepatic impairment, and in patients with concomitant cardiovascular disease. The results of the questionnaire have shown that pazopanib is perceived as an effective drug, in which quality of life (QoL) outcomes are valued above all.

**Conclusions:**

This paper offers a comprehensive and critical summary of efficacy, tolerability, and pharmacokinetics of pazopanib in the treatment of mRCC. Pazopanib is an effective treatment with an acceptable safety profile. Its QoL and tolerability results offer certain advantages when compared with other therapeutic alternatives, and its use appears to be safe in different patient profiles.

## Background

Renal cancer accounts for 2% of all cancers diagnosed in adults, although the incidence is higher in developed countries. Every year, approximately 295,000 new cases are diagnosed, and around 134,000 deaths are recorded [1, 2]. The most common kidney cancer in adults is renal cell carcinoma (RCC), which accounts for 90% of malignant kidney tumors; of these, an estimated 80–85% are clear cell tumors. Other less commonly diagnosed tumors include papillary and chromophobe RCC [3, 4]. Despite curative surgery of the localized tumor, it is estimated that approximately 30% of patients will subsequently develop metastatic disease [5].

Clear cell RCC are highly vascularized tumors that accumulate lipid and glycogen [6–8]. The identification of dysregulated signaling pathways in vascular endothelial growth factor (VEGF) and the mammalian target of rapamycin (mTOR) in the progression of RCC has led to the development and approval of molecules targeting these pathways for therapeutic purposes [9]. Tyrosine kinase inhibitors (TKI) and mTOR inhibitors are now therapeutic options in first-, second- and third-line treatment in patients with metastatic RCC (mRCC) [6, 10–12].

Pazopanib is a multikinase inhibitor which targets the VEGF receptor (VEGFR), the platelet-derived growth factor receptor (PDGFR) and c-Kit protein, inhibiting angiogenesis [13–15]. It is indicated in adult patients with mRCC, both as a first-line treatment and after previous treatment with cytokines [16].

Given the large volume of evidence that is generated year after year in the field of oncology, publications that review and collect the most relevant data help professionals involved in the management of patients to keep informed of the latest developments, so reviews of the literature in oncology, as in other therapeutic areas, are relevant and necessary. On the other hand, it is known that the real world practice is much more complex and variable than the research environment. In addition, clinicians may have different opinions about the relative value of the published data, which may cause several professionals to opt for very different attitudes to face the same problem. Therefore, it could be interesting to have documents containing the review of published data along with expert opinions. This type of combined documents can be useful to know if published evidence is transferred / reflected in routine clinical practice. Moreover, they can help to improve the knowledge and as a consequence of the clinical practice, since they give a global vision both “theoretical” (published data) and “practical” (expert opinion), on a specific topic. Several review papers on the evidence of pazopanib in the first-line treatment of mRCC have been published in the last 5 years [17–23]. However, no single document has included data on the efficacy, tolerability and pharmacokinetics of pazopanib, combined, moreover, with the opinion of an expert group. The aim of this work was to conduct a review of pazopanib and compare it with the clinical practice. This review document presents the available evidence published to date on the three fundamental aspects of pazopanib in the first-line treatment of mRCC: efficacy, tolerability and pharmacokinetics. It also includes a qualitative survey among specialist medical oncologists in renal cancer on certain aspects of standard clinical practice and the management of mRCC patients treated with pazopanib.

## Methods

**Fig. 1:**
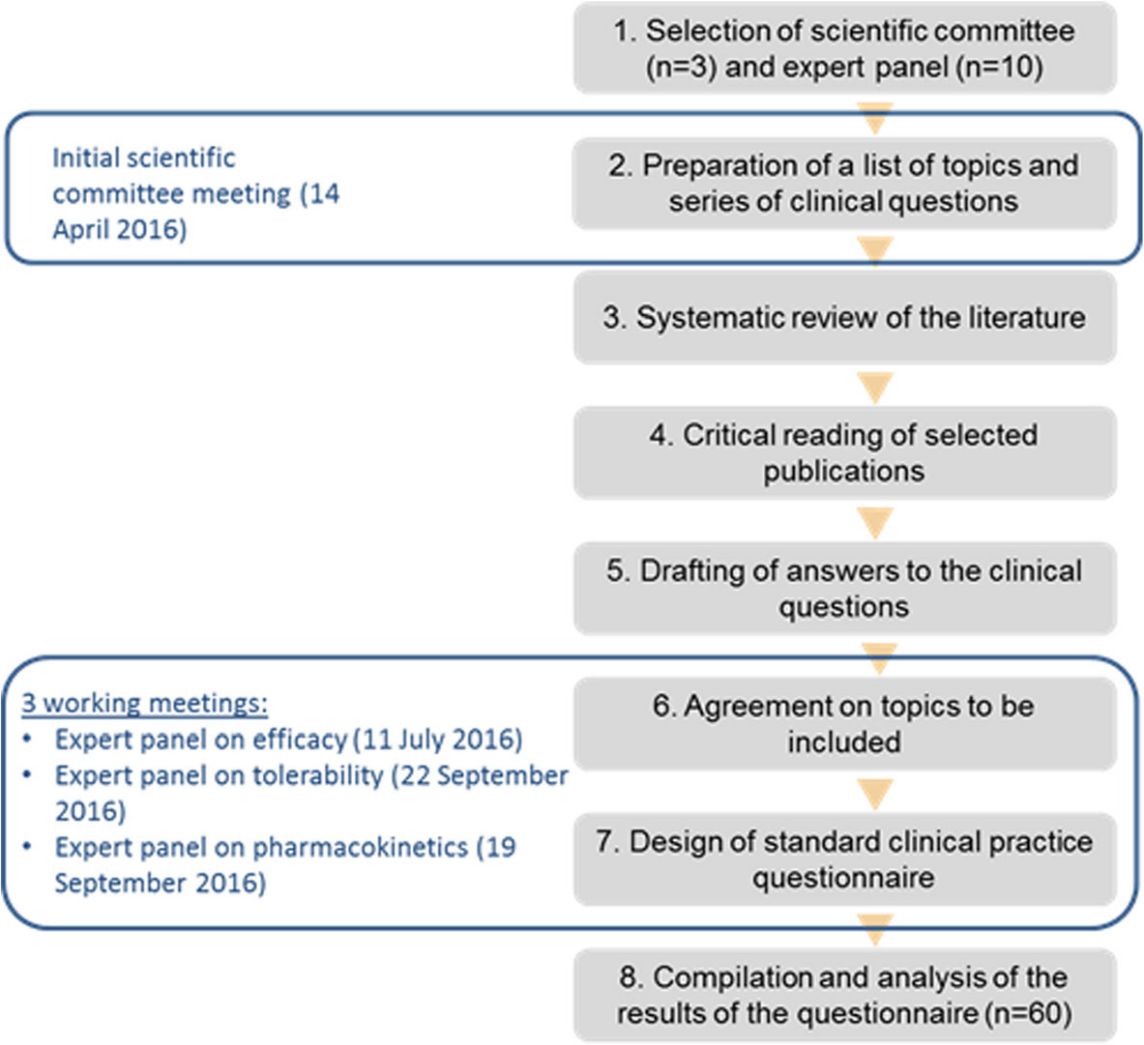
Right 3 Study methodology

The methodology of this review article is summarized in Fig. 1. A scientific committee was formed, consisting of 3 specialist medical oncologists in kidney cancer, who, in an initial in-person meeting led by an expert moderator, agreed on a series of clinical questions. Clinical questions were categorized into 3 main sections: efficacy, safety, and pharmacokinetics of pazopanib. A non-exhaustive systematic review of the literature in Medline was then conducted to identify publications that could answer these questions. Generic key words employed were: “carcinoma, renal cell”[MeSH] AND “pazopanib”[All Fields] AND “humans”[MeSH Terms]. No publication date restrictions were imposed and articles in English or Spanish were included. For the pharmacokinetics section, a specific search was conducted which also included the following key words: “pharmacokinetics” OR “pharmacokinetic” OR “pharmacodynamics” OR “pharmacodynamic” OR “pharmacology”*.* Each member of the scientific committee selected publications of interest for one of the sections. These articles were consulted and synthesized in specific templates. The resulting synthesis of articles was then used by a panel of another 10 specialist medical oncologists in renal cancer to draw up the responses to the proposed clinical questions. These responses were distributed and discussed, and a consensus was reached during 3 in-person meetings; all meetings were attended by a member of the scientific committee and several members of the expert panel. At the same meetings, a questionnaire on aspects of clinical practice related to the evidence on pazopanib in the treatment of mRCC was drawn up consensually (Table 1). The questionnaire was completed by 60 specialist medical oncologists in renal cancer, 68.3% of whom stated that they saw 10 or more mRCC patients a year, and 98.3% of whom had 3 or more years of experience in the area. The completion of the questionnaire was considered implied consent to participate. This document presents the evidence gathered, the conclusions of the expert panel, and the most relevant results obtained from the questionnaire.

**Table 1:** Questionnaire on the first-line use of pazopanib in clinical practice

	Question	Response options
1a	Is achieving a complete response or a partial response with pazopanib in first-line treatment directly correlated with better OS^a^?	1 (strongly disagree) 4 (strongly agree)
1b	Is achieving a complete or partial response with pazopanib in first-line treatment directly correlated with better PFS^b^?	1 (strongly disagree) 4 (strongly agree)
2	Even if there is no impact on OS, is the longer PFS in first-line treatment with pazopanib sufficient for it to be considered as a standard treatment option?	1 (strongly disagree) 4 (strongly agree)
3	Should OS be considered as a primary objective in first-line studies?	a) Yes b) No
4	When selecting pazopanib as a first-line treatment, to which parameter do you give more importance in daily clinical practice?	a) OS b) PFS c) ^c^QoL
5	Does reducing the dose of pazopanib compromise its efficacy?	1 (strongly disagree) 4 (strongly agree)
6	When prescribing a treatment, do you inform the patient about the different first-line treatment options in order to take into account their opinion and preference?	1 (strongly disagree) 4 (strongly agree)
7a	Is pazopanib an appropriate first-line treatment for patients with clear cell carcinoma, ECOG^d^ 0, and no significant comorbidities?	1 (strongly disagree) 4 (strongly agree)
7b	Is pazopanib an appropriate first-line treatment for patients with brain metastases?	1 (strongly disagree) 4 (strongly agree)
7c	Is pazopanib an appropriate first-line treatment for patients with concomitant cardiovascular disease?	1 (strongly disagree) 4 (strongly agree)
7d	Is pazopanib an appropriate first-line treatment for patients with concomitant liver disease?	1 (strongly disagree) 4 (strongly agree)
7e	Is pazopanib an appropriate first-line treatment for patients with non-clear cell histologies?	1 (strongly disagree) 4 (strongly agree)
7f	Is pazopanib an appropriate first-line treatment for patients with moderate to severe renal failure (CrCl^e^ ≤ 30)?	1 (strongly disagree) 4 (strongly agree)
7 g	Is pazopanib an appropriate first-line treatment for patients with ECOG ≥2?	1 (strongly disagree) 4 (strongly agree)
7e	Is pazopanib an appropriate first-line treatment for patients with a poor prognosis?	1 (strongly disagree) 4 (strongly agree)
7f	Is pazopanib an appropriate first-line treatment for patients with asymptomatic heart disease and ^f^LVEF < 50%?	1 (strongly disagree) 4 (strongly agree)
8	When starting treatment with pazopanib in patients treated with oral anticoagulants, what do you do in your standard clinical practice?	a) Change treatment to ^g^LMWH b) Continue ^h^OAC treatment
9a	With regard to patient age and pazopanib treatment, indicate your level of agreement with the following statement: “I do not prescribe pazopanib in patients older than 80 years of age”	1 (strongly disagree) 4 (strongly agree)
9b	With regard to patient age and pazopanib treatment, indicate your level of agreement with the following statement: “In patients over 70 years of age, I prescribe treatment at a dose of 800 mg/day”.	1 (strongly disagree) 4 (strongly agree)
10	If biomarkers for pazopanib response were available, which would be most useful for you in your daily clinical practice?	a) Biomarkers predicting toxicity b) Biomarkers predicting efficacy
11	With regard to tolerability, place in order of importance, from highest to lowest, the following factors to be taken into account when prescribing pazopanib.	a) Functional status b) Concomitant diseases c) Age d) Social support
12	Is it advisable to temporarily suspend treatment with pazopanib, and to continue with radiological monitoring, in order to reduce pazopanib toxicity (stop-and-go strategy)?	1 (strongly disagree) 4 (strongly agree)
13a	If pazopanib toxicity develops, what do you do in the case of grade 2 gastrointestinal toxicity?	a) Maintain the dose b) Reduce the dose c) Discontinue treatment
13b	If pazopanib toxicity develops, what do you do in the case of grade 3–4 gastrointestinal toxicity?	a) Maintain the dose b) Reduce the dose c) Discontinue treatment
13c	If pazopanib toxicity develops, what do you do in the case of grade 1–2 liver toxicity (AST/ALT 2.5 times the normal value)?	a) Maintain the dose b) Reduce the dose c) Discontinue treatment
14	If the patient shows complete response to pazopanib, what do you do in your standard clinical practice?	a) Maintain the dose b) Adjust the therapeutic regimen c) Discontinue treatment

^a^OS: overall survival; ^b^PFS: progression-free survival; ^c^QoL: quality of life; ^d^ECOG: Eastern Cooperative Oncology Group; ^e^CrCl: creatinine clearance; ^f^LVEF: left ventricular ejection fraction; ^g^LWMH: low molecular weight heparin; ^h^OAC: oral anticoagulants

## Results and discussion

### Pazopanib efficacy

#### Progression-free survival

Evidence on the effectiveness of pazopanib in the first-line treatment of mRCC has been obtained from several clinical trials which have shown a progression-free survival (PFS) of between 8 and 11 months, either compared to placebo or to sunitinib [14, 24]. The COMPARZ study demonstrated the non-inferiority of pazopanib compared to sunitinib in terms of PFS. This evidence is consistent with that obtained in a retrospective comparative study [25, 26] (Table 2) and also in the real-life retrospective SPAZO study [25, 26] which showed a PFS of 11.1 months. Other studies have reported PFS rates of between 10.5 and 14.1 months [19, 27–29].

**Table 2:** Pazopanib efficacy data from comparative studies

VARIABLE	STUDY	DRUGS	OUTCOME	STATISTICS
Progression-free survival	VEG105192 [14]	Pazopanib vs placebo	11.1 vs 2.8 months	HR^a^=0.40; 95% CI^b^: 0.27–0.60
	COMPARZ [24] (non-inferiority)	Pazopanib vs sunitinib	8.4 vs 9.5 months	HR = 1.05; 95% CI: 0.90–1.22
	International mRCC Database Consortium (IMDC) [26]	Pazopanib vs sunitinib	8.3 vs 8.3 months	(HR = 1.08; 95% CI: 0.98–1.19)
Response rate	VEG105192	Pazopanib vs placebo	30% vs 3%	Pazopanib: 95% CI: 25.1–35.6Placebo: 95% CI: 0.5–6.4
	COMPARZ (non-inferiority)	Pazopanib vs placebo	31% vs 25%	*p* = 0.003
Overall survival	VEG105192 [14]	Pazopanib vs placebo	22.9 vs 20.5 months	HR = 0.91; 95% CI: 0.71–1.16
	COMPARZ (non-inferiority)	Pazopanib vs sunitinib	28.4 vs 29.3 months	HR = 0.91; 95% CI: 0.76–1.08

^a^HR: hazard ratio ^b^CI: confidence interval;

#### Response rate

Response rate data are listed in Table 2. Response rates based on the independent evaluations performed in the VEG105192 and the COMPARZ studies were significantly superior to those obtained with placebo and sunitinib, respectively. On the other hand, the overall response rate with pazopanib in the SPAZO study [25, 26] was of 30.3%, showing, as in the case of PFS, high consistency with published rates from clinical trials.

Recently, a post hoc exploratory analysis identified and described the clinical characteristics of patients in COMPARZ who exhibited a long-term response to pazopanib or sunitinib (patients with complete response (CR) / partial response (PR) or PFS ≥ 10 and ≥ 18 months), showing PFS ≥ 10 months in 31.4% patients and PFS ≥ 18 months in 14.2% patients, and a shorter time to response with pazopanib compared with sunitinib (11.9 vs 17.4 weeks).

#### Overall survival

Mean overall survival (OS) in the VEG105192 study was not statistically significant (Table 2). This may be explained by the frequent and early switching of patients in the placebo arm to the pazopanib arm, and the inclusion of patients both previously treated and previously untreated with cytokines [14, 30, 31]. In the COMPARZ study, no significant differences in mean OS were observed between pazopanib and sunitinib (Table 2), either in the main study or the latest update: 28.3 vs. 29.1 months, respectively [32]. In various real-world clinical practice studies, a mean OS of 19–40.8 months has been described, probably depending on the proportion of patients with poor prognosis included in the sample [27–29, 33]. Recent studies with the new second-line treatments recommended by the ESMO guidelines, such as nivolumab and cabozantinib, show that after the administration of pazopanib in first line, the median OS for cabozantinib in second line is 22 months, while median OS for nivolumab has not been reached at the time of publication of this manuscript [34–36].

#### Quality of life

In the pre-planned analysis of quality of life (QoL) in the VEG105192 study, no significant differences were observed between the QoL with pazopanib or placebo, as assessed using the EORTC-QLQ-C30 and EQ-5D questionnaires and the EQ-5D visual analog scale [14]. The COMPARZ study also evaluated patient QoL, observing a significant improvement with pazopanib in 11 of the 14 domains that assessed health-related quality of life (HRQoL) during the first 6 months of treatment [24]. In the PISCES study (a patient preference study), HRQoL was evaluated using the Functional Assessment of Chronic Illness Therapy-Fatigue (FACIT-Fatigue) scale: pazopanib obtained a 2.5-point higher score than sunitinib (95% CI: 0.92–4.07; *p* < 0.002). The Supplementary Quality of Life Questionnaire (SQLQ) questionnaire also showed better results with pazopanib in 5 parameters [37, 38]. Finally, in a post-hoc analysis, a trend towards a lower risk of experiencing a > 20% loss of QoL was observed with pazopanib compared to placebo [39].

#### Expert opinion in standard clinical practice

Evidence published on the effectiveness of pazopanib is consistent and positions pazopanib as a standard treatment option. In view of the positive results for pazopanib in terms of OS and PFS, we asked the experts who completed the questionnaire about how these parameters relate with complete or partial response obtained with pazopanib, and how important these parameters are when considering pazopanib as an initial treatment option. In total, 85% of respondents agreed or strongly agreed that the complete or partial response rates achieved with pazopanib are directly correlated with a higher PFS, while 57.5% of respondents associated these parameters with a longer OS. In addition, 95% of respondents agreed that the longer PFS in first line is sufficient to consider pazopanib as a standard treatment option.

Almost 50% of the specialists who responded to the questionnaire stated that they give more importance to QoL as an efficacy parameter than to PFS or OS when selecting pazopanib as first-line treatment. In the case of complete response with pazopanib, 87.5% of respondents stated that they would maintain the therapeutic regimen, while 12.5% would discontinue it.

### Pazopanib tolerability

Most adverse events recorded in the VEG105192 study with pazopanib were grade 1 or 2; 52% of patients had diarrhea; 40% arterial hypertension (AHT); 38% hair depigmentation, 26% nausea; 22% anorexia; and 21% vomiting [14]. The incidence of grade 3 or 4 adverse events was 7% for placebo and 33% for pazopanib, the most common being arterial hypertension and diarrhea.

The safety profiles of pazopanib and sunitinib in the COMPARZ study were significantly different [24] (Table 3). Patients who received pazopanib showed lower incidences of fatigue, hand-foot syndrome, stomatitis, leukopenia, thrombocytopenia, neutropenia, and anemia. No differences were observed in the incidence of cardiovascular adverse events, myocardial infarction or ischemia. In contrast, a higher incidence of elevated alanine aminotransferase (ALT) levels was reported with pazopanib. These differences in side effects between the two drugs are maintained in patients who require dose adjustment during treatment: the incidence of hematologic adverse events and palmar-plantar erythrodysesthesia was higher in the sunitinib group compared to the pazopanib group, whereas scant differences were observed in diarrhea, fatigue, and hypertension [40].

**Table 3:** Percentage of patients with adverse effects observed in the COMPARZ study

ADVERSE EFFECT	PAZOPANIB	SUNITINIB
Fatigue		
Any grade	50%	63%
Grade 3–4	10%	17%
Hand-foot syndrome		
Any grade	29%	50%
Grade 3–4	6%	11%
Stomatitis		
Any grade	14%	27%
ALT^b^	60%	43%
Fatal AE^a^s	2%	3%
Fatal treatment-related AEs	3 cases	8 cases

^a^*AE*: adverse events; ^b^*ALT*: alanine aminotransferase

Safety data from the pivotal studies were subsequently confirmed in real-world clinical practice. Table 4 gathers the main findings regarding to adverse events in three studies. In these studies adverse events were evaluated according to Common Terminology Criteria for Adverse Events (CTCAE). All grades adverse events were recorded in the study by Matrana et al and US cohort study. Only 3 and 4 grades are presented in SPAZO publication. Matrana et al. reported a study in patients treated with pazopanib after progression on first-line targeted treatment, in which 91% of the adverse events were grade 1 or 2, and no treatment-related deaths occurred [28].

**Table 4:** Percentage of patients with adverse effects in real-world studies

Adverse effect	Matrana [28]*	US cohort [33]*	SPAZO [25]**
Fatigue	44%	56%	7.7%
AST^a^/ALT^b^	35%		3.9%/7.8%
Diarrhea	30%	52%	3.6%
Hypothyroidism	18%		
Nausea/vomiting	17%	40%/44%	−/1.1%
Arterial hypertension	–	27%	4%
Anemia			2.6%
Dropout rate	12%		11.9%

^a^*AST* aspartate aminotransferase; ^b^*ALT* alanine aminotransferase

*All grades adverse events according to CTCAE; **Only grade 3–4 adverse effects were reported

Neither deaths due to toxicity nor unexpected toxicities were reported in the Spanish SPAZO study. Although toxicity, especially low-grade toxicity, could have been underestimated as this was a retrospective study [25], the toxicity data for pazopanib in standard clinical practice are lower than those reported in clinical trials. This may due to the growing body of experience among specialists in the management of toxicities associated with renal cancer treatments, confirmed in the American cohort study, in which dose adjustment was reported in 17% of the patients [33], a lower percentage than reported in the COMPARZ study, along with a discontinuation rate of 24% and a mean daily dose of 664.9 mg [24].

#### Special populations

To date, no data have been published on the use of pazopanib in patients with heart disease, although the potential cardiovascular adverse effects of pazopanib should be taken into account when prescribing pazopanib in patients with this profile. In clinical trials, 40–46% of patients had arterial hypertension [14, 24], although the incidence of cardiac dysfunction was less than 0.5–1% [16]. Blood pressure on starting treatment should be lower than 140/90 mmHg and if a patient develops severe hypertension or a hypertensive crisis, antihypertensive therapy should be administered, and the treatment should be suspended and restarted at a reduced dose, according to clinical criteria [16]. No evidence is available in patients treated with oral anticoagulants (OAC), so if treatment is continued, the patient should be monitored closely.

No specific evidence is available on the use of pazopanib in patients with liver disease. Although cases of elevated ALT and AST have been reported, concomitant elevations of alkaline phosphatase or bilirubin are not generally observed [16]. In this population, it is recommended that liver function tests are performed before and during treatment with pazopanib. In case of mild or moderate liver failure (LF), the indications in the summary of product characteristics on administration and patient monitoring should be followed. In patients with moderate LF, the recommended dose is 200 mg pazopanib once a day. However, pazopanib is not recommended in patients with severe LF.

The pivotal trials of pazopanib included patients with renal impairment (RI), but evidence in patients with creatinine clearance (CrCl) < 30 ml/min is limited. Good tolerability and a low incidence of serious adverse events were observed in a retrospective study of 9 patients with end-stage renal disease [41]. Some studies have included patients with mRCC on hemodialysis treated with TKI, in whom pazopanib was safe and effective and increased the life expectancy [42, 43]. Isolated proteinuria is not an indication for dose reduction, unless it is within the nephrotic range (> 3.5 g/day), or presents with edema, hyperlipidemia, or hypoalbuminemia [44].

#### Expert opinion in standard clinical practice

The results of the questionnaire survey show disparate opinions with regard to actions to be taken in the event of toxicity: 45% of the participants were of the view that temporarily suspending treatment with pazopanib could be an option to consider, while the other 55% disagreed. These differences may initially be explained by some vagueness regarding the type or grade of the toxicity in question; however, similar disparity was also observed in successive questions despite a more specific description of the situations. In the case of grade 2 gastrointestinal (GI) toxicity (4–6 bowel movements), half of the respondents stated that they would not reduce the dose, while the other half stated that they would, while for grade 3 or 4 GI toxicity, 57.5% stated that they would reduce the dose and the remaining 42.5% would discontinue treatment. Results also differed in the case of grade 1 or 2 liver toxicity. Lastly, in patients receiving OACs, most experts stated that they would switch treatment to low molecular weight heparins (LMWH), and only 15% would reduce the OAC dose.

It was also noted in the questionnaire results that the patient’s functional status and comorbidities are considered relevant when prescribing pazopanib. Ninety percent of experts considered these factors as quite or very important to take into account. While 65% considered functional status as very important, the presence of comorbidities was considered very important by 30%. Moreover, patient age and available social support were considered as of little or no importance by 90% of the respondents.

### Efficacy and safety of pazopanib in different patient profiles

#### Risk groups

In the subgroup analysis of the COMPARZ study [24], the non-inferiority results for pazopanib compared to sunitinib in terms of PFS were independent of the risk groups, whether evaluated by the Memorial Sloan Kettering Cancer Center (MSKCC) classification [45] or the International Metastatic Renal Cell Carcinoma Database Consortium (IMDC) [46]. According to the MSKCC, mean PFS in low-risk patients with pazopanib was 13.7 months, 8.3 months in intermediate-risk patients, and 3.0 months in high-risk patients [47].

In the SPAZO study, the IMDC risk criteria were validated in first-line treatment with pazopanib, and efficacy and safety were demonstrated in all groups. In total, 19.4% of patients treated with pazopanib had a favorable prognosis, 57.2% intermediate, and 23.4% poor prognosis. Significant differences in PFS were observed in the 3 risk groups: 32.4 months in patients with a favorable prognosis, 11.1 months in the intermediate prognosis group, and 4 months in those with a poor prognosis [25]. In a recent observational study in which 12% of patients had an Eastern Cooperative Oncology Group (ECOG) score of 2 or higher, median PFS and OS were 12.5 and 26.5 months, respectively, in patients with intermediate/favorable risk; and 2.4 and 7.2 months in patients with a high risk, respectively [48].

Recently, an update of SPAZO 2 study results was presented in ESMO 2017. SPAZO 2 is an extension and update of SPAZO 1 trial with a total series of 530 patients in 50 centres treated in 1st line with pazopanib and a median follow up of 39.2 months. Population is somewhat different from SPAZO 1 trial with regard to the proportion of IMDC prognostic subgroups (14.2% favorable, 61.9% intermediate and 24.9% poor prognosis). Results show a median PFS 9.8 months for the whole population, and 19.1, 10.4 and 5.2 months for favorable, intermediate and poor prognosis populations, respectively. Median OS were 19.6 months for the whole population, and 37.2, 20.8 and 7.2 for the favorable, intermediate, and poor prognosis populations, respectively. Relative risks were 32.9 for the whole population, and 58.9, 35.2 and 22.5% for the favorable, intermediate and poor prognosis populations, respectively. In this study, eligibility for clinical trials was tested as a prognostic factor. The study confirms the effectiveness of pazopanib in mRCC in the real world setting and concludes that ineligibility might be considered as a prognostic factor for PFS and OS (HR: 1.35; 95% CI 1.05–1.73) [49].

Drug tolerability was not related with prognostic groups in any of the studies reviewed. However, the results of the observational studies which included patients with a worse prognosis or with ECOG 2 or higher [48] are similar to those obtained in the VEG105192 [14] and COMPARZ clinical trials [24].

#### Age

In registrational studies, 33% of the patients were older than 65 years of age, 3% were over 75, and patients aged up to 83 years of age were recruited [14, 16]. Elderly patients were also included in the COMPARZ and PISCES studies [24, 37]. Although no conclusions can be drawn due to the small number of elderly patients included in these trials, there do not appear to be any differences in effectiveness compared to sunitinib, and the safety profile among patients receiving pazopanib was similar, regardless of their age.

#### Functional status and other factors

In general, treatments show better PFS outcomes in patients with good prognosis, ECOG performance status of 0, and a period of 1 year or more between the initial diagnosis and treatment for metastatic disease [50]. In the subgroup analysis of the COMPARZ study, PFS outcomes were similar for both treatments, regardless of the ethnicity, geographical origin or Karnofsky performance status of patients [24]. Although no specific analyses have been conducted, several observational studies have included patients with poor prognostic variables (ECOG > 2, patients diagnosed less than 1 year previously, 3 or more MSKCC risk factors or 3 or 4 IMDC risk factors, histologies other than clear cell renal carcinoma, brain metastases, advanced age, or comorbidities), in which pazopanib has shown efficacy results consistent with those published in clinical trials [25, 29, 51, 52].

#### Expert opinion in standard clinical practice

**Fig. 2:**
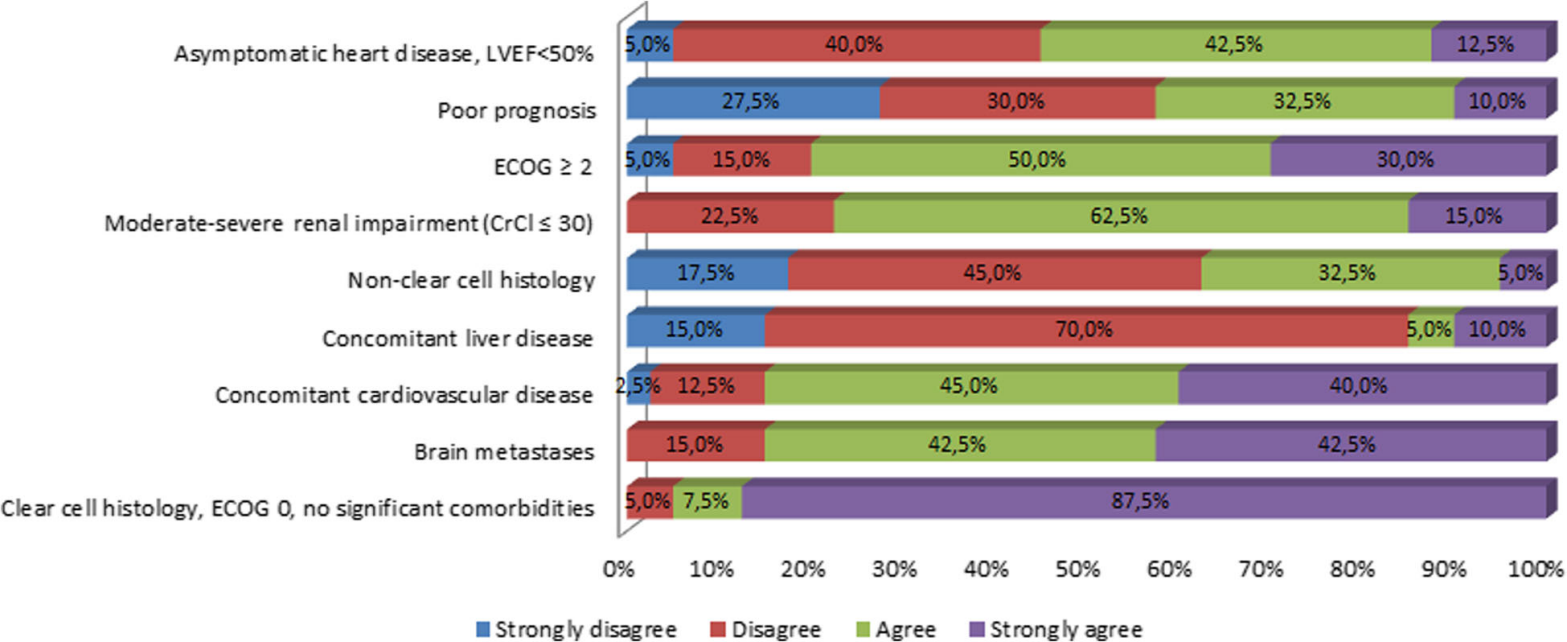
Results of the questionnaire on the use of pazopanib in standard clinical practice according to patient profile (CrCl: creatinine clearance; ECOG: Eastern Cooperative Oncology Group: LVEF: left ventricular ejection fraction)

Pazopanib has shown clinical benefit in all risk groups, regardless of age, tumor histology, location of metastases, and functional status or ECOG score. In this respect, the experts completing the questionnaire were asked how they perceived the appropriateness of using pazopanib in different patient profiles, depending on comorbidities, prognosis, site of metastases, or functional status. In total, 95% of respondents felt that pazopanib is a suitable option for the treatment of patients with clear cell histologies, no comorbidities, and ECOG 0. Between 80 and 85% of respondents agreed that pazopanib was appropriate for use in the first-line treatment of patients with brain metastases, cardiovascular comorbidities, or ECOG of 2 or greater. Almost 80% saw pazopanib as a first-line treatment in patients with moderate-to-severe RI. Although, as outlined above, the available evidence is insufficient to advise against or contraindicate the use of pazopanib in the following cases, more than half of participants did not consider it as an appropriate first-line treatment in patients with concomitant liver disease, non-clear cell histology, or poor prognosis (Fig. 2). With regard to the appropriateness of using pazopanib in elderly patients, almost all respondents considered that patients over the age of 80 years are candidates for treatment with pazopanib, and almost 90% agreed that doses of 800 mg/day could be prescribed in patients older than 70 years. This opinion was consistent with the populations of patients included in clinical trials and observational studies, which may reassure clinicians’ performance in this specific group of patients.

### Efficacy and safety biomarkers and polymorphisms

#### Biomarkers

The discovery of biomarkers can be of use in predicting inter-patient differences in the efficacy and safety of antineoplastic agents and for identifying patients who are resistant to treatment. If the right drug is given to the right patient, the chances of response might increase, or the appearance of certain adverse reactions might be predicted, helping to achieve greater therapeutic success rates. Unfortunately, identifying these markers in the early stages of clinical development of the drugs remains a challenge, and they are generally identified when the medications have been administered to more extensive patient populations.

Arterial hypertension has been considered a biomarker of response to angiogenesis inhibitors [53]. In the case of pazopanib, a subanalysis of the COMPARZ study noted a trend (not statistically significant) towards a longer PFS in patients with a higher grade of systolic hypertension in weeks 4 and 12 of treatment (*p* = 0.06 and 0.07, respectively), as well as a trend towards longer OS in patients with higher systolic hypertension in week 4 (*p* = 0.062) [54].

High baseline plasma concentrations of interleukin (IL)-6, IL-8, osteopontin and VEGF have been associated with an increased response to pazopanib in terms of OS, while high baseline levels of hepatocyte growth factor (HGF) have been associated with a lower OS in patients receiving pazopanib [55]. In another publication, high plasma concentrations of IL-8, osteopontin, HGF and tissue inhibitor of metalloproteinases 1 (TIMP1) were associated with a lower PFS in patients receiving pazopanib, while IL-6, IL-8 and osteopontin were also associated with a lower PFS in the placebo group. This latter finding might suggest a relationship between these 3 markers and RCC progression, rather than an association with response to pazopanib [56]. A recent subanalysis in patients in the COMPARZ study found that a mutation in the PBRM1 suppressor gene is associated with a better response to TKI, in the form of increased PFS and OS, as it seems to enhance the pro-angiogenic microenvironment of RCC. In contrast, the BAP1 mutation is associated with decreased angiogenic signaling and a poorer outcome for TKIs, manifesting as a shorter OS [57].

#### Polymorphisms

VEGF genotypes s833061TT, rs2010963CC, and rs699947CC have been associated with a greater clinical benefit in patients receiving pazopanib [58]. Polymorphisms in the IL-8 gene and hypoxia-inducible factor 1A (HIF-1A) have been associated with PFS in pazopanib-treated patients, and polymorphisms in the HIF-1A, nuclear receptor subfamily 1, group I, member 2 (NR1I2), and VEGFA genes have been associated with response rates [59]. The rs1126647 genotype of the IL-8 gene has been associated with a shorter OS [60] and the variant rs34231037 in the VEGFR2 gene has been associated with greater sensitivity to pazopanib treatment [61].

The UGT1A1 TA-repeat polymorphism (Gilbert’s syndrome) is significantly associated with increased bilirubin levels in patients treated with pazopanib [62]. TA7/TA7 homozygous or TA6/TA7 heterozygous patients can show an increased risk of developing hyperbilirubinemia when receiving pazopanib [63]. The VEGFR2 gene + 1416 T > A (His472Gln) polymorphism has been associated with increases in blood pressure during treatment with pazopanib, particularly in AA homozygous patients. Similar results were observed with the VEGFA gene -2578C > A, -1498C > T and -634G > C polymorphisms [64]. Specific polymorphisms such as the rs2858996/rs707889 in the HFE gene may be associated with reversible ALT elevation in patients treated with pazopanib [65].

#### Expert opinion in standard clinical practice

The identification of biomarkers that can predict response to antiangiogenic therapy may be of vital importance to mRCC patients. At present, no biomarkers that predict response to treatment with pazopanib have been validated prospectively, and as such, these data cannot be applied to clinical practice. In the questionnaire, 95% of respondents felt that if biomarkers were identified, those predicting efficacy would be more useful than those which predicted safety.

### Pazopanib pharmacokinetics

According to the phase I study in patients with solid tumors treated with pazopanib, maximum plasma concentration (C_max_) and area under the curve (AUC_0–24_) on day 1 increased as the pazopanib dose increased, up to the maximum dose of 2000 mg/day. However, steady-state exposure is achieved at a dose of pazopanib 800 mg/day [66].

Pazopanib is extensively bound to plasma proteins (99.9%), regardless of its plasma concentration [67], and it is primarily excreted as unchanged drug in feces.

In the phase I study, a minimum plasma concentration (C_min_) of 15 mg/l or more was associated with a partial response [66]. A C_min_ of > 20.5 mg was subsequently associated with a greater reduction in tumor size (37.9% vs. 6.9% with a C_min_ < 20.5 mg/l) and a longer PFS (52 weeks vs. 19.6 weeks with a C_min_ < 20.5 mg/L) [68].

The greatest variability among patients treated with pazopanib is caused by the ingestion of fat in the diet, as this factor may double the AUC or C_max_ of the drug. Therefore, it is recommended that pazopanib is administered 1 h before or 2 h after meals. Other factors, such as age, race or gender, do not seem to be related to inter-patient variability [69, 70]. The use of concomitant medications that alter gastric pH is also associated with inter-patient variability [68]. Genetic variability may also affect the response to pazopanib, although as noted above, no validated biomarkers are currently available. The concomitant administration of cytochrome P450 CYP3A4 inhibitors and pazopanib results in an increase in plasma concentrations, because pazopanib is a substrate of CYP3A4 and, to a lesser extent, CYP1A2 and CYP2C8 [71]. In contrast, CYP3A4 activity may be enhanced in patients with the NR1/2 T allele, resulting in increased pazopanib clearance [59].

The pazopanib dose of 800 mg daily was selected on the basis of the phase II and III study data, plasma concentrations associated with the clinical and biological effects in preclinical models and in patients with solid tumors [72, 66], and because the steady-state plasma concentration of 20.5 μg/ml is associated with longer PFS and reductions in tumor size. Even so, pazopanib adverse events are dependent on plasma concentration [68], so the dose sometimes needs to be reduced, initially to 600 mg and then to 400 or 200 mg, if necessary [16]. In this context, it has been observed that the percentage of patients within the target window during fixed dosing is not significantly different from that of patients whose doses are adjusted [73]. On the other hand, a subanalysis of the COMPARZ study noted a longer PFS with both drugs in patients requiring a dose adjustment for the management of toxicities, suggesting that dose reductions or temporary treatment interruptions do not affect efficacy [40].

#### Expert opinion in standard clinical practice

If adverse effects appear, adjusting the pazopanib dose reduces toxicity and does not appear to compromise efficacy. In contrast to reports, the perception of most experts surveyed (62.5%) is that reducing the pazopanib dose compromises efficacy.

## Conclusions

This paper offers a comprehensive and critical summary of three important aspects of pazopanib in the treatment of mRCC: efficacy, tolerability, and pharmacokinetics. It also includes data on special populations and a summary of potential future biomarkers. The efficacy results of pazopanib in terms of PFS, OS and response rates are consistent across all studies.

Pazopanib is a treatment with an acceptable safety profile. Its QoL and tolerability results offer certain advantages when compared with other therapeutic alternatives, and its use appears to be safe in different patient profiles, including the elderly and patients with heart disease, mild or moderate liver failure, or poor functional status. Before pazopanib is indicated, the patient must be fully assessed, taking into account their functional status, comorbidities, and concomitant medications, rather than simply assessing patient age, thus minimizing toxicities. The first dose reduction in the event of adverse effects with pazopanib is from 800 mg to 600 mg, and this adjustment does not appear to compromise efficacy. Intra- and inter-patient variability is mainly associated with gastric pH and food intake.

The questionnaire allowed us to compare the available evidence on the use of pazopanib with the perceptions of the drug among a group of medical oncologists, all experts in the treatment of RCC. The results of the questionnaire have shown that pazopanib is perceived as an effective drug, in which QoL outcomes are valued above all.

After reviewing the efficacy and safety data of the drug, this paper concludes that pazopanib can be used in patients of any prognostic group, functional status, and age. Observational studies also show that pazopanib is effective in patients with non-clear cell histology, brain metastases, and in different metastatic sites, although the opinion of the experts consulted may vary, as in the case of patients with liver diseases. In view of the above, we can conclude that the experts view pazopanib as a good first-line treatment option in the majority of patients with mRCC.

## Abbreviations

AHT: Arterial hypertension
ALT: Alanine aminotransferase
AST: Aspartate aminotransferase
AUC0–24: Area under the curve
Cmax: Maximum plasma concentration
Cmin: Minimum plasma concentration
CR: Complete response
CrCl: Creatinine clearance
CTCAE: Common Terminology Criteria for Adverse Events
ECOG: Eastern Cooperative Oncology Group
FACIT-Fatigue: Functional Assessment of Chronic Illness Therapy-Fatigue
GI: Gastrointestinal
HGF: Hepatocyte growth factor
HIF-1A: Hypoxia-inducible factor 1A
IL: Interleukin
IMDC: International Metastatic Renal Cell Carcinoma Database Consortium
LF: Liver failure
LMWH: Low molecular weight heparins
mRCC: Metastatic renal cell cancer
MSKCC: Memorial Sloan Kettering Cancer Center
mTOR: Mammalian target of rapamycin
OAC: Oral anticoagulants
OS: Overall survival
PDGFR: Platelet-derived growth factor receptor
PFS: Progression-free survival
PR: Partial response
QoL: Quality of life
RCC: Renal cell carcinoma
RI: Renal impairment
SQLQ: Supplementary Quality of Life Questionnaire
TIMP1: Tissue inhibitor of metalloproteinases 1
TKI: Tyrosine kinase inhibitors
VEGF: Vascular endothelial growth factor
VEGFR: VEGF receptor
